# Establishing a telemedical supported trans-sectoral collaboration network from community support to emergency care for outpatient care recipients: study protocol of a prospective non-randomized complex intervention study with a pragmatic approach, Stay@Home – Treat@Home

**DOI:** 10.1186/s12877-024-05553-6

**Published:** 2024-12-02

**Authors:** Doreen Müller, Deborah Elisabeth Jachan, Maria Stahl, Marie-Luise Rosenbusch, Daniela Liersch-Mazan, Peggi Lippert, Niels Hinricher, Maria Ivanova, STH Research Group, Mandy Schulz, Nils Lahmann

**Affiliations:** 1 Central Research Institute of Ambulatory Health Care (Zi), Department of Epidemiology and Healthcare Atlas, Salzufer 8, Berlin, 10587 Germany; 2 Charité – Universitätsmedizin Berlin, Corporate member of Freie Universität Berlin and Humboldt Universität zu Berlin, Institute of Medical Sociology and Rehabilitation Science, Charitéplatz 1, Berlin, 10117 Germany; 3 Charité – Universitätsmedizin Berlin, Corporate member of Freie Universität Berlin and Humboldt Universität zu Berlin, Department of Geriatrics and Medical Gerontology, Nursing Research Group in Geriatrics, Augustenburger Platz 1, Berlin, 13353 Germany; 4 Berlin Institute of Health at Charité – Universitätsmedizin Berlin, QUEST Center for Responsible Research, Charitéplatz 1, Berlin, 10117 Germany; 5 HCMB – Institute for Health Care Systems Management Berlin eG, Amorstraße 29, Berlin, 12526 Germany; 6 Central Research Institute of Ambulatory Health Care (Zi), Department of Healthcare Analysis, Salzufer 8, Berlin, 10587 Germany; 7 MSB Medical School Berlin, Rüdesheimer Str. 50, Berlin, 14197 Germany

**Keywords:** Outpatient care recipients, Telemedicine, Digital interactive health diary, Trans-sectoral care, Study protocol

## Abstract

**Background:**

Demographic changes in Germany are increasing the number of outpatient care recipients, who often resort to emergency care due to difficulties accessing timely outpatient medical care. Previous studies suggest that early detection and telemedical interventions could reduce unnecessary hospitalizations. The new form of healthcare aims to provide continuous, flexible healthcare for outpatient care recipients using digital technologies to detect health deteriorations and facilitate interventions at home. The goal of our study is to evaluate, whether the number of emergency situations and hospital stays will be reduced, and health outcomes will be improved compared to standard care.

**Methods:**

In this prospective non-randomized complex intervention study with a pragmatic approach, we aim to evaluate a new form of healthcare focused on establishing an interdisciplinary network for outpatient care in the homes of care-dependent individuals. Utilizing a digital interactive health diary, health data will be gathered from participants, caregivers, and healthcare providers, covering both stable primary care and acute situations. A telemedical network will coordinate measures, including non-medical aid, nursing care, and medical assistance. A total of 1,500 participants will be recruited for the intervention group, matched with a control group from health insurance data. A second control group with n=300 will provide self-reported measures. The study is planned to span eight quarters, with data collected from the digital interactive health diary and health insurance records. Evaluation perspectives include health insurance, patients, and healthcare providers, assessing utilization and costs compared to standard care, health status, health-related quality of life, care dependency, interdisciplinary cooperation, and usability of the new technology.

**Discussion:**

Demographic change results in a larger older people population, exacerbating mobility issues and care dependency, worsened by the shortage of medical personnel. Stay@Home – Treat@Home aims to enable home health monitoring and care, reducing hospitalizations. The digital interactive health diary supports direct communication, allows remote monitoring, and empowers patients and caregivers to manage health changes. Nursing aid personnel and physicians can access entries for informed interventions. The development of the digital interactive health diary aims to improve the situation of care-dependent individuals. Evaluating its effectiveness and efficiency is crucial for the development and implementation of new technologies.

**Trial registration:**

German Clinical Trials Register, ID: DRKS00034260, registered on May 14, 2024 (retrospectively registered): https://drks.de/search/de/trial/DRKS00034260 and https://who.int/clinical-trials-registry-platform/network/who-data-set.

## Introduction

### Background

Due to demographic changes, the number of outpatient care recipients is increasing nationwide in Germany. In the year 2050, approximately one in ten people in certain regions of Germany will be dependent on care [1]. Four out of five are cared for at home, and more than half of them is 80 years or older [2]. People who require care often experience impairment in their health and mobility [3]. As a result, they require regular medical supervision but face greater challenges in accessing outpatient medical care, particularly specialist medical services [2]. Another area of concern is the critical use of medication. Every 7th person in need of care receives a prescription for medication with associated risks. Additionally, polypharmacy is often a result of uncoordinated healthcare and more common among those in need of care, posing a risk of adverse drug interactions [2].

Consequently, people who are care dependent rely on emergency services and inpatient care more frequently, resulting in a high number of unplanned and sometimes avoidable hospital admissions [4, 5]. On average, every care dependent person in Germany is hospitalized twice a year [2]. Particularly among older care recipients with cognitive impairments, this exacerbates prognosis dramatically, leading to increased mortality, longer hospital stays, and a higher risk of rehospitalization [6–9]. Furthermore, the transfer from home to the hospital can facilitate significant psychological distress for those affected, known as relocation stress or transfer trauma [10, 11].

Previous studies have shown that at least 30 percent of multimorbid care recipients in emergency departments would not require inpatient therapy if health changes were detected early on and timely medical assessment and treatment, for instance through telemedicine, could be arranged [12, 13]. Furthermore, interdisciplinary coordination of healthcare measures is beneficial for health outcomes.

###  Objective

The aim of the intervention is to provide continuous, needs-based, trans-sectoral and flexible healthcare for individuals receiving care at home during episodes of illness and health crises. Within the trans-sectoral care network established for this purpose, a new digital technology is intended to enable the rapid detection and communication of deteriorations in health status and facilitate early intervention in the home environment. The aim of this study is to assess whether the implementation of a telemedical supported trans-sectoral collaboration network can lead to a reduction in emergency situations and unplanned hospital admissions, improve state of health, quality of life, and care needs of individuals compared to standard care, and to evaluate the enhancement of interdisciplinary cooperation and usability facilitated by the new technology.

##  Methods

###  Design

This is a prospective non-randomized complex intervention study with a pragmatic approach [14, 15]. Important protocol modifications are decided within the project consortium, then submitted to the project sponsor. Subsequently, changes to the German Clinical Trials Register entry will be made.

###  Intervention

The new form of healthcare Stay@Home – Treat@Home (STH) focuses on establishing an interdisciplinary and trans-sectoral network for low-threshold outpatient care in the home of care dependent individuals. This is facilitated through the digital interactive health diary, a telemedical application. Here, information about the participants' health status is collected by themselves, their caregivers, their general practitioner, and all stakeholders involved in acute care within the new form of healthcare and is accessible by them when needed [16]. The new form of healthcare covers two areas of care: primary care as well as acute and emergency care.

The primary care takes place as long as participants are in a stable health condition without acute care needs. Their health status is regularly recorded and reviewed by themselves, their caregivers, and their primary care physician within the digital interactive health diary.

**Fig. 1:**
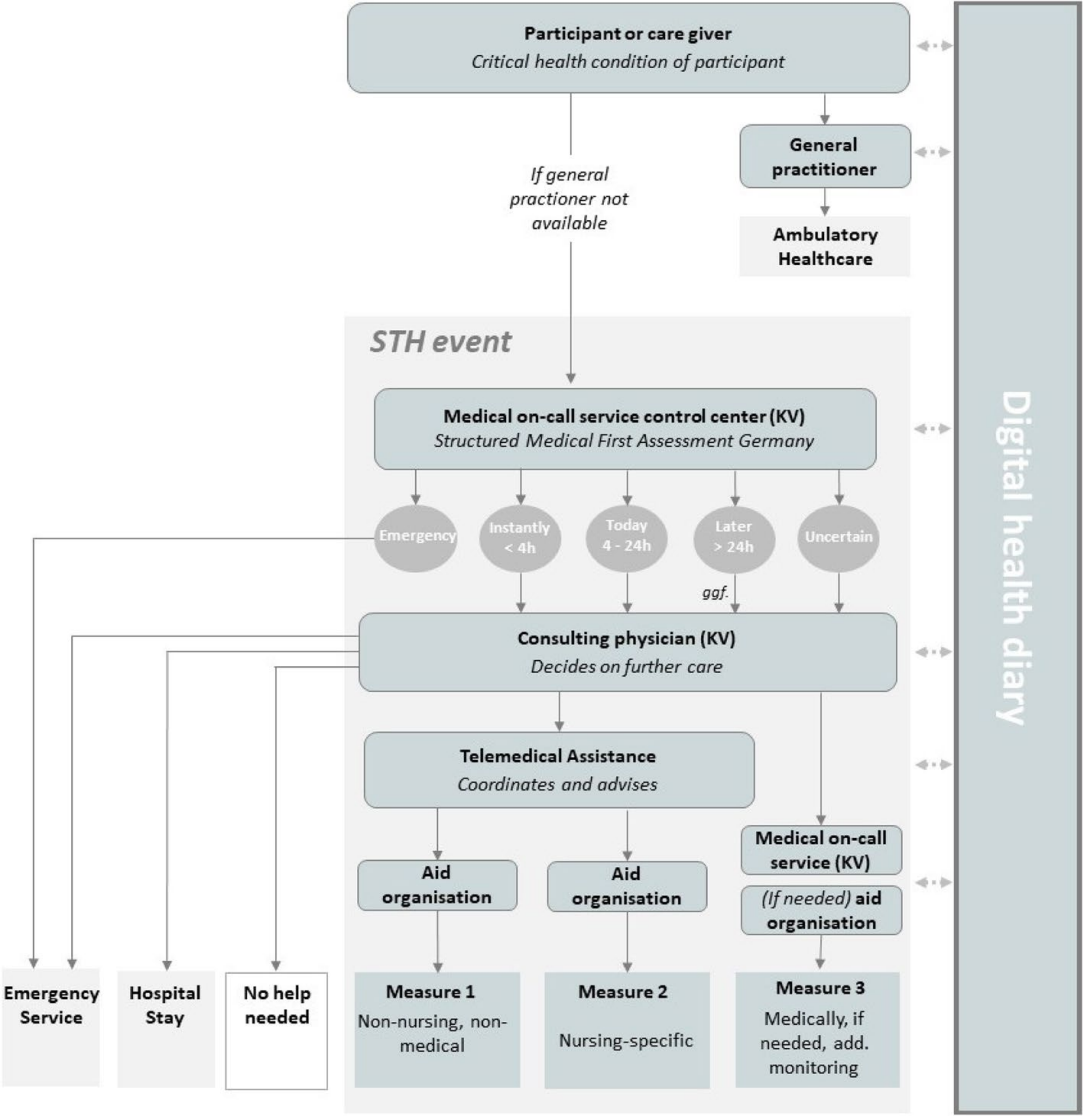
Care pathways of the new form of healthcare (simplified representation). STH event: event within the new form of healthcare; KV: Association of Statutory Health Insurance Physicians

Acute and emergency care comes into play when regular entries in the digital interactive health diary enable the early detection of deteriorating health conditions. Initially, participants or their caregivers contact their general practitioner (GP), who then provides care to the participants as part of standard care. If the general practitioner is unreachable, participants call the medical on-call service of the Berlin Association of Statutory Health Insurance Physicians (KV Berlin) using a special hotline stored in the digital interactive health diary, thereby activating the care network. This triggers an STH event. The medical on-call service center determines the current care needs based on the symptoms of the patient/participant as well as their entries in the digital interactive health diary using the Structured Initial Medical Assessment in Germany (SmED) [17]. The care pathways are outlined in Fig. 1.

If immediate medical intervention is required, an emergency is triggered, the emergency medical services are alerted, and the STH event is terminated. If the case is not an emergency, and it is clearly determined that measure 1 or 2 is required, the event is passed on to the telemedical assistance at the Charité. Otherwise, the medical on-call service of the association contacts the association's consulting physician, who then decides on further care for the participants. There are three possibilities: transfer of the case to standard care with simultaneous termination of the STH event (emergency use or referral to the hospital; light grey boxes in Fig. 1); closing of the case, as no acute assistance is necessary (white box in Fig. 1); or an intervention within the framework of the new form of healthcare (dark grey boxes in Fig. 1). Three types of interventions in the participants' home environment are provided: Measure 1 contains a general assistance provided by a nursing aid service without any further medical or nursing assistance. Measure 2 consists of nursing intervention offered by a nursing aid service or the participants' home care service. Measure 3 entails medical intervention facilitated by the medical on-call service, potentially combined with monitoring by a nursing aid service or remote treatment through the telemedical assistance.

While the consulting physician directly assigns the task for measure 3 to the mobile medical on-call service of the association, measures 1 and 2 are controlled via the telemedical assistance, to which the consulting physician assigns the case. Additionally, during an STH event, the telemedical assistance can be utilized by all involved care providers at any time for consultative and coordinating activities. All stakeholders can also access information about the participants' health status in the digital interactive health diary, and all care steps are recorded by the stakeholders in the digital interactive health diary. Every STH event is reviewed and acknowledged by the participants' general practitioner, enabling therapy adjustments for the patients in the primary care setting, if necessary.

Adherence will be also encouraged through regular communication and guidance by the general practitioners as part of the study protocol. There will be no formal follow-up conducted after the intervention. However, reasons for participant discontinuation will be systematically collected to understand the factors influencing dropout rates.

###  Participants

 Assuming that the hospitalization rate in the patient group before the intervention is approximately 20 percent [2] with a potential of reduction to 15 percent during intervention (a reduction of 25 percent), with a power of 90 percent and a significance level of 0.05, the calculated sample size is 1,210 patients for the intervention group [18]. To address sample attrition due to heightened mortality in the research group as well as other unforeseeable drop-outs, about 25 percent additional patients should be recruited, resulting in a total sample size of *n* = 1,500 participants in the intervention group, with at least the same number of controls to be drawn. GPs will be recruited through outreach by the KV, which in turn will recruit eligible participants for the study. Alternatively, the participants will be recruited by a study physician to relieve the GPs. The informed consent will be obtained from the participants by either their GP or the study physician. The intervention group will be derived from eligible insurees of the participating health insurance companies, who are 60 years or older, reside in Berlin in their own home, receive support from a caregiver, and have been either officially granted “care level” 1 or higher, have applied for it or been assessed by their GP as needing “care level” 1 or higher. In Germany, five “care levels” represent a scale used to classify the level of care needed by individuals requiring long-term care. The respective care level determines the subsidies those individuals receive through their nursing care insurance provider. Participants can withdraw their consent to participate in the study anytime. If participants stop meeting the inclusion criteria listed above at some point, e.g. leave their participating insurance company, they will be excluded from the study.

For the control group from the health insurance data (control group 1), insured individuals with similar characteristics to those in the intervention group will be identified from the enrollee and billing data of the participating health insurance companies. For better comparability, the aim is for the control group to also originate from Berlin, Germany. However, if the numbers are not sufficient, patients from structurally similar settlement areas will be included in the control group. A minimum 1:1 matching is planned, ensuring that the control group will be the same size as the intervention group. Depending on the number of insured individuals in the participating health insurance companies, a 1:n matching may also be performed, thereby increasing the size of the control group accordingly.

The control group for the secondary outcomes (control group 2) will be *n* = 300.

The rationale of this study is to select control groups that do not receive the intervention and an intervention group that does. It is based on the study's objective to assess the efficacy of the intervention in comparison to no treatment. For the control group 2 individuals with similar characteristics to those in the intervention group, but being insured by other than the participating health insurance companies, will be included in the study. This design allows it to determine the direct impact of the intervention by observing differences between the groups. The use of two no-intervention control groups is ethically justifiable in this context, as the expected benefits of the intervention are hypothesized but not yet established. This approach ensures that any observed effects can be attributed specifically to the intervention, thereby providing clear insights into its potential benefits.

###  Study period

**Fig. 2:**
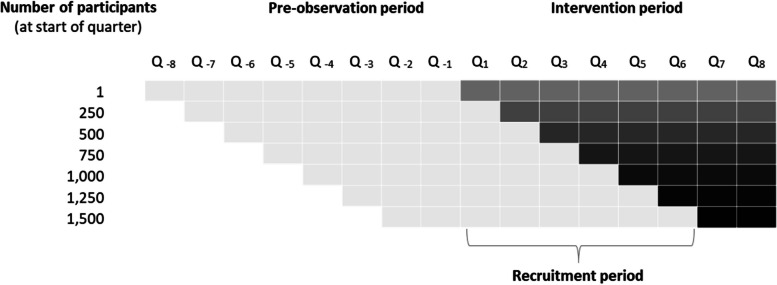
Planned data collection periods

The study period is designed to take place within eight quarters (24 months). During the first six quarters (18 months), the intervention group will be gradually recruited for the new form of healthcare, so that individuals who are added at the end of the recruitment period can utilize the new form of healthcare for at least two quarters (6 months). For the control group 1 and the operationalization of control variables, the claims data of the health insurance companies from a pre-observation period of eight quarters before entry into the new form of healthcare will be used. The surveys on the secondary endpoints take place at the beginning of the intervention (weeks 2-4) and after 3 to 6 months during the intervention. The control group 2 is surveyed at a congruent time interval. Figure 2 illustrates the planned data collection period and the cumulatively increasing sample size of the intervention group.

###  Data sources

The data for the evaluation will be gathered from two main data sources. Firstly, various reports will be generated from the digital interactive health diary. These reports will encompass health-related basic data entered by participants, their caregivers, and their primary care physicians, such as current condition, diagnoses, therapies, medication plans, level of care needed, allergies, and measured vital parameters. Additionally, questionnaires for process evaluation and evaluation of health and quality of life will be collected via the digital interactive health diary as well as via the online platform SoSci Survey. Furthermore, all data for the STH event will be extracted through digital interactive health diary reports for the evaluation of utilization patterns and costs, as well as for process evaluation.

As a second data source, data from the health insurance companies will be utilized (enrollee data, claims data concerning sick pay, nursing care, short-term care, outpatient and inpatient cases, ambulance services, prescription of medicinal products, medical devices, and aids). This will be used to establish the control group 1 and to obtain data for comparing the new form of healthcare with standard care.

The reports from the diary are continuously accessible in pseudonymized form by the evaluating parties. Each evaluation party receives only the data necessary for their specific research questions. For the evaluation utilization patterns and costs, it is necessary to merge the data from the diary with the enrollee and billing data from the health insurance companies. This is because treatments provided by the mobile medical on-call service within the framework of STH events are partly reimbursed and documented through STH project funds and partly through statutory health insurance.

Data collection forms cannot be provided here. However, all variables and their response formats are described within the protocol.

###  Data Monitoring

The present study does not require a Data Monitoring Committee (DMC) due to its design and the nature of the intervention. The study involves a low-risk intervention with well-established safety profiles and outcomes. The potential for adverse events is minimal, therefore the need for ongoing oversight is not justified. Instead, regular internal oversight measures to ensure adherence to protocol and monitor data quality throughout the study will be implemented.

These are also the reasons, why this study does not require formal auditing of trial conduct. The internal oversight mechanisms in place will adequately ensure that the trial is conducted according to established guidelines and standards.

###  Data Storage

Personal information about potential and enrolled participants will be collected through secure, anonymized data collection methods, ensuring that identifiers are removed whenever possible. Data will be stored in password-protected databases with restricted access to authorized personnel only. During the trial, any sharing of personal information will be limited to essential personnel involved in the research, and all data will be shared in aggregated or de-identified form whenever feasible. A trusted third-party organization will be involved to link data while maintaining confidentiality and ensuring that sensitive information is protected. After the trial, all personal information will be maintained in a secure location for a designated period, and then permanently deleted, in compliance with data protection regulations.

###  Outcomes

Within the evaluation, three perspectives will be considered: health insurance, patients, and health care providers. All outcome variables are shown in Table 1.

**Table 1:** Outcomes

**Outcome**	**Operationalization**	**Data source**	**Outcome type**
**Health insurance perspective**			
Utilization: Hospital stays and emergency responses	Number of hospital stays/ emergency responses	Claims data	primary
Utilization: Ambulatory-care sensitive hospital stays and emergency responses	Ambulatory-care sensitive diagnoses	Claims data	primary
Utilization: ambulatory resource allocation	Ambulatory medical utilization	Claims data	secondary
Cost: New form of healthcare	All costs directed towards the new form of healthcare	Diary and claims data	primary
Cost: Comparison of new form of healthcare versus standard care	Comparison with saved costs of standard care	Diary and claims data	primary
**Patient perspective**			
Health	QoL-AD; own design of questions	Diary data and SoSci Survey	secondary
Well-being	WHO-5; OSSS-3; UCLA-LS	Diary data and SoSci Survey	secondary
Care dependency	Barthel-Index	Diary data and SoSci Survey	secondary
**Healthcare provider perspective**			
Interdisciplinary cooperation	Own design of questions	Diary data	secondary
Suitability of tasks	Own design of questions	Diary data	secondary
Usability	BGW	Diary data	secondary

#### Health insurance perspective

On the level of the health insurance, utilization and total costs will be compared between intervention group and control group 1. Concerning utilization, number of hospital stays, days spent in inpatient hospital care, and number of emergency situations will be analyzed. For these measures, a second analysis will focus on ambulatory-care sensitive hospital stays and emergency responses [13, 19]. Furthermore, the volume of healthcare provided by the outpatient sector will be evaluated. For the economic evaluation, the total costs of the new form of healthcare in addition to costs for standard care within the intervention group will be assessed and compared to the costs of standard care for the control group 1 [20–22].

The rationale for selecting the number of treatments (e.g. hospital stays) and the overall treatment costs as the primary outcome domains is based on their direct relevance to the study’s objectives and clinical impact. The number of treatments provides a quantifiable measure of intervention utilization, offering insights into the intensity and frequency of care required. Meanwhile, total treatment costs are essential for evaluating the economic implications of the intervention, which is particularly important for healthcare systems focusing on cost-effectiveness and resource allocation. Together, these domains allow for assessing both the practical and financial viability of the intervention, making them highly pertinent to stakeholders and policymakers.

#### Patient perspective

 On the patient level, health, well-being, and care dependency will be evaluated in a pre-post-design, using data from the intervention group as well as the control group 2 (*n* = 300). Seven questions compiled on the basis of clinical experience will be used to assess general health. The participants of the intervention and control group 2 will be asked weekly about falls, pain, urinary excretion, restlessness, shortness of breath, dizziness, and an open-ended question about any health problems experienced within the last seven days. The Quality of Life-Alzheimer’s Disease (QoL-AD) [23–26] is used to assess cognitive functioning in week 2-4 and 3-6 months after starting participation. Within 13 items, it covers physical health, energy, mood, living situation, memory, family, marriage, friends, chores, joy, money, own self, and life in general. QoL-AD possesses a good content, criterion concurrent and construct validity, as well as an excellent interrater reliability (Cohen's κ > 0.70) and internal consistency (Cronbach's α = 0.82) [24].Quality of life will be assessed using the Wellbeing measures in primary health care (WHO-5) questionnaire, containing five questions about mental well-being, like being in a good mood, feeling energetic, or being interested in daily life [27–29]. WHO-5 has satisfactory psychometric properties, both in terms of reliability (e.g. internal consistency α =.92, split-half reliability *r*_*tt*_= .87), construct and factorial validity [29]. The Oslo social support scale (OSSS-3) will be used to assess social support through three questions about relationships with close individuals and the availability of neighborly help [30, 31]. Its predictive and construct validity is reported to be good, while internal consistency (α = .640) is acceptable [30]. Moreover, the UCLA Loneliness Scale is added to measure loneliness within 20 items, evaluating topics like social isolation, companionship, and perceived social support [32]. Existing validation studies come to favorable assessments with regard to criterion, convergent and discriminant construct validity, as well as reliability (retest reliability >.70, internal consistency α =.89, split-half reliability *r*_*tt*_=.85) [32]. The WHO-5 questionnaire, OSSS-3 and the UCLA Loneliness Scale will be conducted in week 2-4 as well as 3-6 months after beginning to participate.

Care dependency will be assessed using the Barthel-Index [33, 34]. Here, ten activities of daily living are assessed regarding the capability of conducting them independently. Participants will conduct the assessment in week 2-4 and 3-6 months after enrolment into the study.

#### Health care provider perspective

On the health care provider level, aspects concerning interdisciplinary cooperation, suitability for the task, and usability will be assessed. According to the cooperation model, successful interdisciplinary cooperation requires support for three subprocesses: coordination, communication, and knowledge integration [35]. These topics will be evaluated using custom-designed questionnaires and interaction protocols derived from the diary.

To assess interdisciplinary cooperation, interaction protocols will be used to examine the content and timing of care during an STH event. For this purpose, all interactions of the respective stakeholders are recorded and stored by the digital interactive patient diary and analyzed using process mining methods [36]. The analysis will be initially carried out every 3 months. As the frequency of events increases, the evaluation will be switched to monthly.

Regarding the suitability/effectiveness of process and task integration, the adequacy of communication support among healthcare providers through the digital interactive health diary, and its facilitation of knowledge integration will be evaluated using a questionnaire. Additionally, we will assess the extent to which the digital interactive health diary assists users in task execution (suitability for the task) using our own designed questions. These evaluations will focus on the timely detection of health status deviations, the accuracy of their urgency and relevance assessment, and the successful assistance of patients during emergencies. Every three months, every stakeholder (the patients, caregivers, and healthcare providers) will receive two questions on communication/knowledge integration and two questions on task appropriateness in the diary, which are adapted to their subtasks in the STH program. In addition, following an STH event, the stakeholders are asked to evaluate the course of care during an STH event. In case of a low rating, possible causes (text modules for selection and free text field) are recorded. The results will help us to identify possible weaknesses at an early stage and to rectify them promptly with suitable measures.

The usability of the digital interactive health diary will be assessed quarterly using a tailored questionnaire by the German Social Accident Insurance Institution for the Health and Welfare services (BGW) [37]. It evaluates suitability, ease of use, long-term adoption potential, workflow and technical adaptation, preference for healthcare with the digital interactive health diary, and clarity of screen content.

###  Analysis

#### Health insurance perspective

Concerning the perspective of health insurance, the control group 1 will be matched through propensity score matching, incorporating sociodemographic variables such as age, gender, level of care dependency, region, as well as morbidity and healthcare utilization in the 12 to 24 months prior to the study commencement. Analyses will focus on differences in the utilization of inpatient and emergency services between the intervention and control groups. Moreover, the study will assess ambulatory-sensitive utilization of inpatient and emergency services. Total costs for the new form of healthcare in addition to costs for standard care will be calculated and compared to the cost savings of standard care. The statistical analyses will be descriptive and inferential, with regression models selected based on data requirements. Additionally, a cost-effectiveness analysis will be conducted.

#### Patient perspective

For the analyses on the patient level, the development of general health, care dependency and well-being will be evaluated over the period of the study (pre-post-comparison) both descriptively and inferentially. Regression models will be selected based on data requirements. Age, female gender, and lower educational attainment [38] are associated with increased morbidity [39], thus these variables will be included as covariates. The control group 2 follows the same inclusion criteria as the intervention group regarding age, level of care dependency and region.

#### Health care provider perspective

The analysis from the health care provider perspective will focus on analyzing the (work) processes of the new form of healthcare, especially the processes during an STH event, and will address issues relating (interdisciplinary) cooperation between stakeholders and the usability of the digital interactive health diary. Process mining techniques will be used to analyze the processes during an STH event as they take place. The results will be compared to the processes as they should be and will identify patterns, inefficiencies, and bottlenecks within the healthcare provider's operations [35]. Descriptive methods will be used to analyze the data collected on (interdisciplinary) collaboration and usability.

There will be an internal interim evaluation from all the three perspectives after half of the intervention time passes. The results will be shared with the participating institutions.

##  Discussion

Demographic change leads to a larger population of older individuals and consequently more people facing mobility issues and care dependency. Furthermore, the shortage of nursing staff and other medical personnel will exacerbate the situation [40]. New forms of care are needed to facilitate the work of healthcare providers and improve the health of care recipients. Advances in medicine and technology can help maintain or even improve the quality of life among the growing older people population [41]. With Stay@Home – Treat@Home, care dependent individuals can be monitored for health at home and provided medical services. Supported by a closely coordinated care network spanning both outpatient and inpatient sectors, and leveraging state-of-the-art technologies and telemedicine, the aim of the study is to evaluate whether this approach leads to a reduction in hospital admissions [42] and alleviate the burden on the healthcare system [43].

The digital interactive health diary serves various functions. Firstly, it facilitates direct communication among patients, their caregivers, and healthcare providers. Regular entries related to health queries and measurements are guided and monitored by the GP. The GPs can access the health documentation stored by patients and caregivers at any time, allowing to remotely monitor the patient's condition. Secondly, patients and their caregivers can more easily detect any changes in health status themselves and take appropriate measures [44]. Thirdly, should an intervention be necessary at the patient's home, personnel of nursing aid services as well as physicians can also access the entries to gain an understanding of the patient's health trajectory. They can document the measures they have taken, thereby providing a record of the course of events for all other healthcare providers involved.

If the digital interactive health diary is to be included in regular standard care, it should not only be beneficial for the patients’ health and cost effective for health insurances. It also has to meet the needs of its direct users. In Germany, the documentation burden for healthcare providers is quite high [45]. This is why this evaluation study considers not only the perspective of the patients and the health insurance, but also evaluates the experiences of the healthcare providers using this new digital interactive health diary [46]. This way, Stay@Home – Treat@Home could potentially take a significant step forward in the advancement of medical and nursing care.

###  Limitations

The approach to recruitment or data access for the intervention and control group 1, respectively, will be different, which can cause different selection bias in the intervention and control group 1.

A further aspect of the study, which contributes to the selection bias is, e.g. the fact that the study will only be conducted in Berlin. Therefore, regional differences cannot be considered and the generalization of the results to rural areas must be approached with caution. Only participants with a caregiver or members of participating health insurance companies can be included in the study, which may also lead to a certain selection bias. However, part of the bias is taken into account by including persons who are insured with a health insurance company that is not participating in the project in control group 2 with regard to the survey on the secondary endpoints. Additionally, for ethical and practicality reasons, a randomized controlled trial cannot be implemented.

There is also a possibility of a measurement bias, if the billing data is not maintained carefully by the responsible organizations. A response bias is possible in the data coming from the digital interactive health diary.

###  Strengths

The strengths of the study include its high ecological validity, as this study is conducted under real world conditions within the frame of real healthcare services in Germany.

## Trial status

Start of recruitment was March 25, 2024, and will continue until June 30, 2025. The observation period will end on December 31, 2025. By the time of submission (November 17, 2024), *n*= 36 participants have already been enrolled in the trial. The results will be reported to the funder, as well as shared with the scientific community and the public in a publication.

Authorship eligibility guidelines within the study consortium are established to ensure that all contributors who meet the criteria for authorship are appropriately credited in publications. Principles of intellectual contribution and accountability in the authorship process are prioritized. There are no intended uses of professional writers for the preparation of manuscripts; instead, all publications will be collaboratively developed by the research team members.

## Data Availability

No datasets were generated or analysed during the current study.

## Abbreviations

BGW: German Social Accident Insurance Institution for the Health and Welfare services
G-BA: German Federal Joint Committee
GP: General practitioner
KV: Association of Statutory Health Insurance Physicians
OSSS-3: Oslo social support scale
SmED: Structured Initial Medical Assessment in Germany
STH: Stay@Home – Treat@Home
QoL-AD: Quality of Life-Alzheimer’s Disease
UCLA-LS: University of California Los Angeles-Loneliness Scale
WHO-5: Wellbeing measures in primary health care
